# Pro-arrhythmic role of adrenergic spatial densities in the human atria: An in-silico study

**DOI:** 10.1371/journal.pone.0290676

**Published:** 2023-08-25

**Authors:** Karl Magtibay, Stéphane Massé, Kumaraswamy Nanthakumar, Karthikeyan Umapathy

**Affiliations:** 1 Biomedical Signal and Image Processing Laboratory, Faculty of Engineering and Architectural Science, Toronto Metropolitan University, Toronto, Ontario, Canada; 2 Toby Hull Cardiac Fibrillation Management Laboratory, Department of Medicine/Cardiology, Toronto General Hospital, University Health Network, Toronto, Ontario, Canada; 3 Biomedical Signal and Image Processing Laboratory, Department of Electrical, Computer, and Biomedical Engineering, Faculty of Engineering and Architectural Science, Toronto Metropolitan University, Toronto, Ontario, Canada; National Institute of Environmental Health Sciences National Toxicology Program Division, UNITED STATES

## Abstract

Chronic stress among young patients (≤ 45 years old) could result in autonomic dysfunction. Autonomic dysfunction could be exhibited via sympathetic hyperactivity, sympathetic nerve sprouting, and diffuse adrenergic stimulation in the atria. Adrenergic spatial densities could alter atrial electrophysiology and increase arrhythmic susceptibility. Therefore, we examined the role of adrenergic spatial densities in creating arrhythmogenic substrates in silico. We simulated three 25 *cm*^2^ atrial sheets with varying adrenergic spatial densities (ASD), activation rates, and external transmembrane currents. We measured their effects on spatial and temporal heterogeneity of action potential durations (APD) at 50% and 20%. Increasing ASD shortens overall APD, and maximum spatial heterogeneity (31%) is achieved at 15% ASD. The addition of a few (5% to 10%) adrenergic elements decreases the excitation threshold, below 18 *μA*/*cm*^2^, while ASDs greater than 10% increase their excitation threshold up to 22 *μA*/*cm*^2^. Increase in ASD during rapid activation increases *APD*_50_ and *APD*_20_ by 21% and 41%, respectively. Activation times of captured beats during rapid activation could change by as much as 120 ms from the baseline cycle length. Rapidly activated atrial sheets with high ASDs significantly increase temporal heterogeneity of *APD*_50_ and *APD*_20_. Rapidly activated atrial sheets with 10% ASD have a high likelihood (0.7 ± 0.06) of fragmenting otherwise uniform wavefronts due to the transient inexcitability of adrenergically stimulated elements, producing an effective functional block. The likelihood of wave fragmentation due to ASD highly correlates with the spatial variations of *APD*_20_ (*ρ* = 0.90, p = 0.04). Our simulations provide a novel insight into the contributions of ASD to spatial and temporal heterogeneities of APDs, changes in excitation thresholds, and a potential explanation for wave fragmentation in the human atria due to sympathetic hyperactivity. Our work may aid in elucidating an electrophysiological link to arrhythmia initiation due to chronic stress among young patients.

## Introduction

Prolonged sympathetic hyperactivity due to disease or injury could result in diffuse neurocardiac remodeling of the atrial myocardia and create arrhythmogenic substrates. Injury and disease are well-known factors of diffuse neurocardiac remodeling as demonstrated in animal models [1, 2]. Previous works have also shown that patients with myocardial ischemia could have neurocardiac remodeling and become vulnerable to arrhythmias [3, 4]. A study by Zhu et al. demonstrated that sympathetic nerves at an ischemic border could potentially have different physiological mechanisms than those distant from a scarred site [5].

Sympathetic hyperactivity due to autonomic dysfunction could create arrhythmogenic myocardial substrates [6, 7]. Hyperactivity of the sympathetic nervous system (SNS) could be coupled with the withdrawal of the parasympathetic nervous system (PNS) [7]. Similarly, hyperactivity of the SNS due to chronic stress could lead to acute cardiac injury [8] or affect DNA methylation in cardiac genes [9] as demonstrated in previous murine studies. Sympathetic hyperactivity is associated with increased circulating catecholamines (i.e., adrenaline and noradrenaline), enlarged neural cell bodies, enhanced synaptic transmission, and dispersed neural innervation of the atria [10]. Sympathetic hyperactivity could result in an exaggerated adrenergic response of atrial tissues, shortening its overall atrial effective refractory period (AERP), and the appearance of early after depolarizations (EAD) in its action potentials (APs) [11, 12]. Shortening of AERP and appearance of EADs due to excess intracellular *Ca*^2+^ mobilized by adrenergic neurotransmitters could be indicators of arrhythmic vulnerability [6].

Prolonged rapid pacing, simulating sympathetic overdrive, has been shown to promote sympathetic nerve sprouting and diffuse increase in sympathetic and parasympathetic innervation in normal canine hearts [13–15]. Previous works on normal canine hearts by Chang et al. [15] on the right atria (RA) and Akira et al. [16] on the left atria (LA) showed that prolonged rapid stimulation promotes heterogeneous atrial sympathetic hyperinnervation. Similarly, chronic psychosomatic stress has been shown to promote autonomic dysfunction and could contribute to neurocardiac remodeling and subsequent arrhythmias in non-ischemic murine models of Post Traumatic Stress Disorders (PTSD) [9, 17, 18]. A heterogeneous increase in atrial sympathetic nerve densities has also been observed in animal and human models of atrial fibrillation (AF) [14–16, 19]. In addition, patients with a history of persistent AF have been shown to have a higher density of adrenergic fibers in their myocardia than patients with only paroxysmal or post-operative AF [11]. However, the electrophysiologic link between sympathetic hyperactivity and atrial arrhythmias remains unclear [20].

The role of spatial densities of sympathetic neuroeffector junctions in human AF initiation is unexplored. Although AF is more common in the elderly with underlying substrate remodeling, young persons (≤ 45 years old) who are less likely to have remodeled atrial tissues could also develop AF due to autonomic dysfunction [21]. Sympathetic hyperactivity due to PTSD in young patients without cardiomyopathies has also been shown to promote AF [22]. Increased risk for AF in PTSD patients may be attributed to electrical remodeling of the atria due to chronic changes in autonomic tone. Findings from previous animal studies may explain the observations by Rosman et al. [22] that young patients with PTSD without underlying cardiac substrate modifications were more likely to develop AF earlier than those without PTSD. Rosman et al. further suggested that AF in the young may be influenced more by psychological factors that alter the electrophysiological characteristics of atrial tissues.

Our study attempts to elucidate a possible electrophysiological link between sympathetic hyperactivity via adrenergic spatial densities and arrhythmia initiation due to sympathetic nerve sprouting in the human atria. We hypothesize that varying adrenergic spatial densities (ASD) affect the spatial and temporal heterogeneity of atrial action potential durations (APDs). We also hypothesize that the presence of adrenergic elements could change the excitation thresholds of the atria. Our work may aid in understanding the electrophysiological relationships between sympathetic hyperactivity and arrhythmic susceptibility in the atria. Our potential findings could aid in developing effective therapies to minimize the likelihood of AF initiation among chronically distressed individuals, especially in young patients. We limit our study to the atria due to its close anatomical relation to the autonomic nervous system.

Our paper is organized as follows. First, we present our methods to simulate and analyze the effect of varying ASDs in atrial sheets by gradually increasing activation rates and external transmembrane currents. Then, we present the results of our simulations, measurements, and statistical analysis. We discuss how our findings relate to the current literature on AF initiation and its implications on initiating AF due to sympathetic hyperactivity.

## Methods

We simulated sympathetic hyperactivity in the human atria via beta-adrenergic activation using the Grandi atrial model [23] in bidomain, as made available in the open cardiac electrophysiology simulator (openCARP) [24]. Transmembrane currents in a bidomain model are expressed as a function of ionic (*I*_*ion*_) and capacitive currents ($C_{m}\frac{\partial V}{\partial t}$) over a cell membrane surface. Transmembrane currents are expressed as functions of intra- (***σ***_***i***_) and extracellular (***σ***_***e***_) conductivity tensors, transmembrane voltage (∇*V*_*m*_), such that

$$\begin{array}{c} \begin{array}{cc} \nabla \cdot \sigma_{i}\left(\nabla V_{m}+\nabla \phi_{e}\right) & =\chi (C_{m}\frac{\partial V_{m}}{\partial t}+I_{ion}-I_{T})\\ \nabla \cdot (\left(\sigma_{i}+\sigma_{e}\right)\nabla \phi_{e}) & =-\nabla \cdot (\sigma_{i}\nabla V_{m}) \end{array} \end{array}$$ (1)

where *χ* is the membrane surface-to-volume ratio, and *C*_*m*_ is the capacitance of the cell membrane. *V*_*m*_ is defined by the difference between intra- (*ϕ*_*i*_) and extracellular (*ϕ*_*e*_) potentials and *I*_*T*_ is the external transmembrane current. We direct the reader to the previous works of Clayton et al. [25] and Vigmond et al. [26] for a detailed explanation of the variables used and the derivation of the above formulas.

The Grandi atrial model in openCARP simulates beta-adrenergic effects via known interactions of isoproterenol in the atria. The Grandi atrial model allows us to focus our study on the effects of ASDs without the variabilities that could be introduced with isoproterenol doses. While alpha-adrenergic receptors are also present in the myocardium [27, 28], electrophysiological effects are governed by the beta-adrenergic response of the atria [29]. Our current work attributes adrenergic effects in the atria only to the beta-adrenergic response. Thus, we refer to the beta-adrenergic activity as the adrenergic response for the remainder of our current work.

The Grandi atrial model simulates the steady-state effects of an adrenergic response as follows: a three-fold increase in conductance and a 40-mV leftward shift from the peak of the current-voltage curve of the slowly activating delayed rectifier *K*^+^ channel; a three-fold increase in conductance of the ultra-rapid delayed rectifier *K*^+^ channel; 50% increase in the fraction of channel availability and leftward shift by three mV for L-type *Ca*^2+^ channel; enhanced *Ca*^2+^-sensitivity of SERCA and ryanodine receptors by 50% and 200%, respectively; the affinity of troponin I to *Ca*^2+^ decreased by 50%; and, decreased affinity of *Na*^+^ pump to intracellular *Na*^+^ by 25%.

We created three 5 cm by 5 cm atrial sheets of quadrilateral mesh elements at 0.5 mm spatial resolution. We simulated an isotropic media, keeping longitudinal and transversal conductances equal (*σ*_*L*_ = *σ*_*T*_ = 0.1617 S/m) to maintain a 40*cm*/*s* conduction speed and minimize the directional effect myocardial fibers. We pre-paced our atrial sheet at approximately 3 Hz with a 2-ms 100 *μA*/*cm*^2^ external transmembrane current using a uniform planar wavefront spanning its left edge to excite elements of the atrial sheet evenly.

### Spatial density of adrenergic stimulation

**Fig 1:**
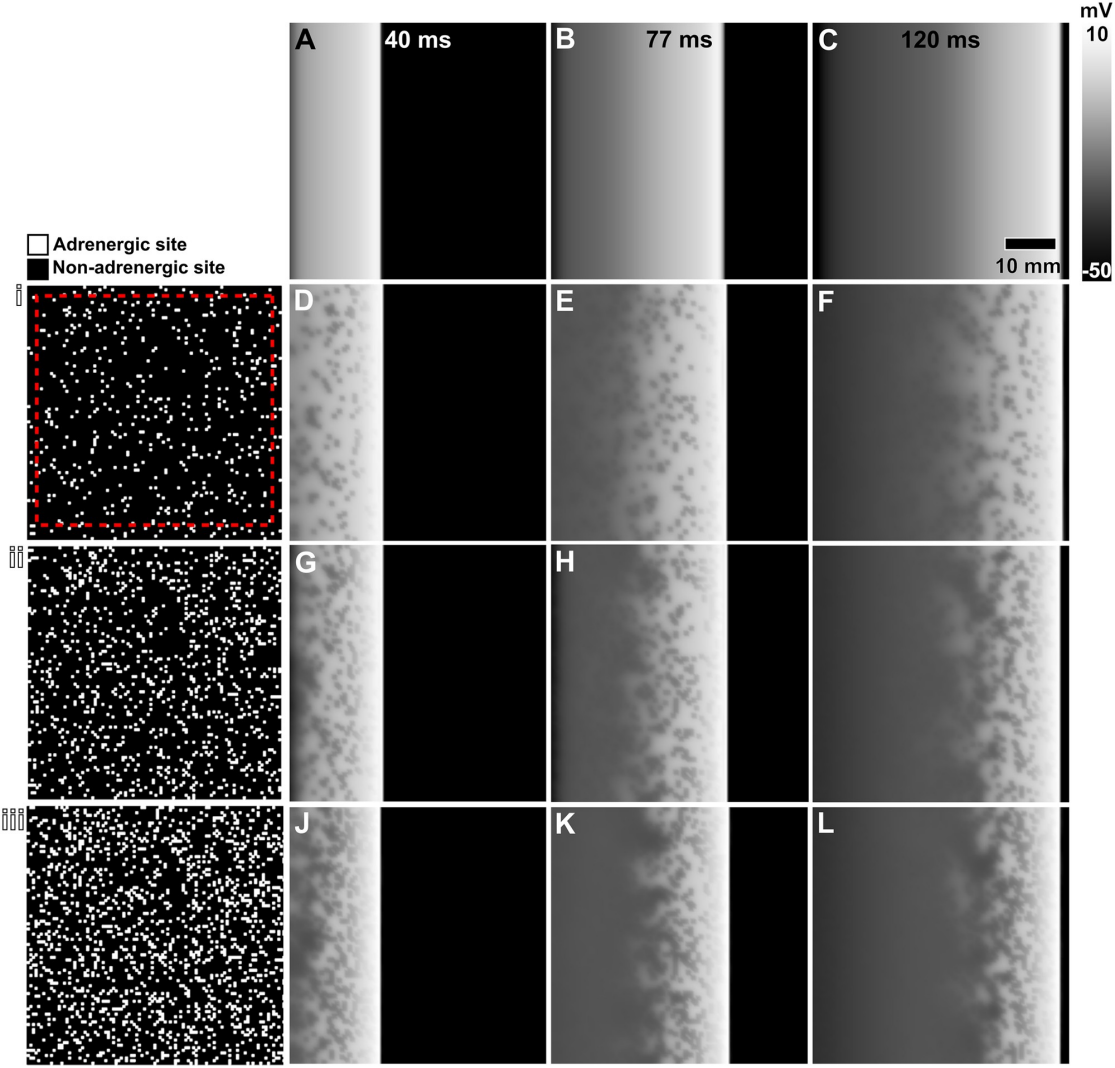
Wave propagation in atrial sheets with increasing adrenergic spatial density. We show gradually increasing adrenergic spatial densities at 5% (**i**), 10% (**ii**), and 15% (**iii**). Black and white mesh elements represent non- and adrenergically stimulated sites, respectively. The red dashed square on (**A**) indicates the area of atrial AP analysis. Comparison of wave propagation at three different time points between baseline atrial sheet(**A**-**C**) and adrenergically stimulated atrial sheets with increasing spatial densities at 5% (**D**-**F**), 10% (**G**-**I**), and 15% (**J**-**L**).

We defined ASD as the ratio of randomly assigned non- and adrenergically stimulated mesh elements. We gradually increased the number of ASD by 5%, starting from 5% (1.25 cm^2^) up to 20% (5 cm^2^). Our ASD range is based on the previous findings by Chang et al. [15] and Akira et al. [16], where the maximum density of sprouted sympathetic nerves was approximately 17% for a given atrial tissue slice. We repeated gradual increments of ASD on three atrial sheets with unique adrenergic distributions. We followed the same stimulation protocol described above for each atrial sheet for at least six beats (approximately 2 seconds). Fig 1i–1iii examples of atrial sheets with increasing ASDs.

We examined the effect of ASDs by measuring action potential durations (APDs) to estimate repolarization durations. APDs were estimated from the duration of the last AP above 90% (*APD*_90_), 50% (*APD*_50_), and 20% (*APD*_20_) of its maximum amplitude. We also measured the spatial variations of AP repolarization via coefficients of spatial variation (CoSV) to examine the effect of ASD on the spatial heterogeneity of APs. CoSV is calculated as the ratio of the standard deviation ($\sigma_{APD_{X}}$) and mean ($\mu_{APD_{X}}$) of APDs of the last beat across an atrial sheet as

$$\begin{array}{c} CoSV_{APD_{X}}=\frac{\sigma_{APD_{X,s}}}{\mu_{APD_{X,s}}} \end{array}$$ (2)

*X* is the APD type of interest (i.e., 90, 50, or 20) and *s* indicates the calculation of values over space. CoSV value that approaches 0% indicates the minimum variation from average APD values. In contrast, a CoSV value of 100% indicates maximal variation. We included data only from mesh elements within the red dashed square in Fig 1i to exclude border and activation artifacts.

### Adrenergic spatial densities, excitation threshold, and activation rates

For each atrial sheet with increasing ASDs, we systematically increased external transmembrane currents from 14 to 22 *μA*/*cm*^2^ to study the effects of ASDs on the atrial excitation threshold. The range of external transmembrane currents was empirically chosen based on the least external transmembrane current required to activate a single cell based on the Grandi atrial model in the bidomain. Moreover, we simulated burst-pacing protocols that promote sympathetic hyperinnervation and AF based on previous works [15, 16, 30]. We pre-paced (at 3 Hz) our atrial sheets for at least two beats using the above stimulation protocol. Then, we burst-paced our atrial sheets for 1.5 seconds using combinations of the above external transmembrane currents and activation rates from 10 Hz to 23 Hz.

We accounted for potential changes in the excitation threshold with ASD. We also analyzed the effect of increasing activation rates on atrial APDs using the abovementioned procedure. Like CoSV, we calculated the coefficients of temporal variation (CoTV) for each APD type to measure the temporal effects of increasing activation rates and ASD.

$$\begin{array}{c} CoTV_{APD_{X}}=\frac{\sigma_{APD_{X,t}}}{\mu_{APD_{X,t}}} \end{array}$$ (3)

*X* is the APD type of interest (i.e., 90, 50, or 20) and *t* indicates the calculation of values over time. Lastly, we measured the absolute activation time difference (|Δ|) by subtracting the average cycle length of captured beats from our baseline cycle length of 300 ms. We define a captured beat as an AP directly resulting from applying sufficient external transmembrane current regardless of activation rate. We considered only AP data from a random mesh element we observed along the conduction path.

### Statistical analysis

We present our statistical measurements as mean and standard error. Due to the nested nature and repeated measures of our APD data, we used a Generalized Linear Mixed Model (GLMM) to analyze the effect of ASD and activation rates on the mean atrial APDs (i.e., *APD*_90_, *APD*_50_, and *APD*_20_), CoSV, and CoTV across three atrial sheets, all at *α* = 0.05. We used a binary logistic regression link with GLMM to calculate probabilities of conduction or the likelihood of tissue excitation relative to external transmembrane currents and ASDs. All statistical analyses were done with SPSS, a proprietary statistical package by IBM [31].

## Results

### Spatial density of adrenergic stimulation

**Fig 2:**
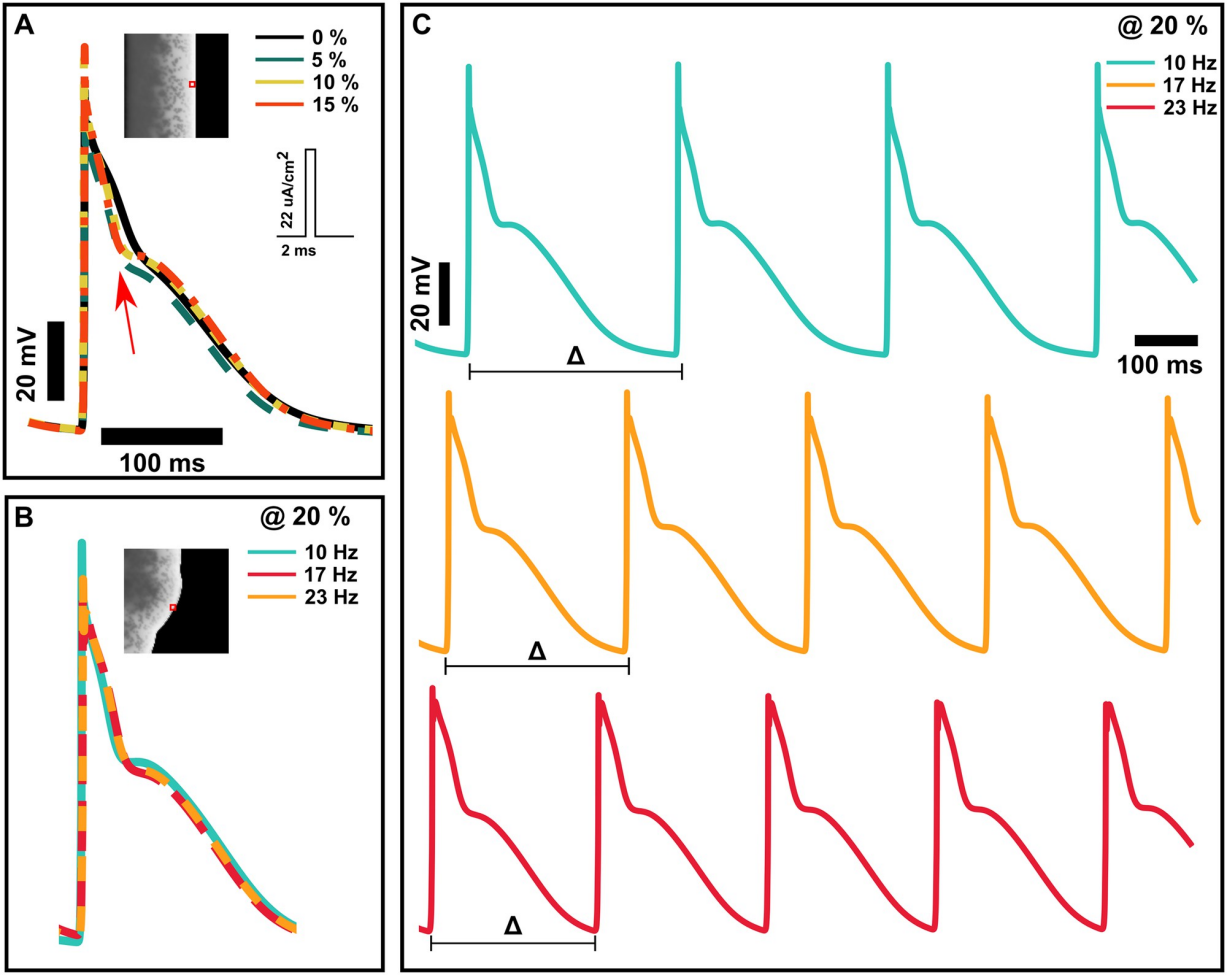
Atrial action potentials from varying adrenergic spatial densities and activation rates. (**A**) Compared to a baseline atrial action potential, a gradual increase in adrenergic spatial density from 5%, 10%, and 15% marginally increases action potential amplitude. It promotes an isopotential phase halfway through repolarization. (**B**) Increased activation rates in the presence of adrenergic elements also contribute to changes in action potential duration characteristics but are not as drastic as adrenergic stimulation alone. (**C**) Increased activation rates in the presence of adrenergic elements produce temporal variations in action potential duration characteristics and change the baseline cycle length of captured beats. The location of the sample action potential on the atrial sheet is highlighted in red boxes.

Results obtained from the analysis of *APD*_90_ were unremarkable. Therefore, we will focus our analysis on the *APD*_50_ and *APD*_20_. We illustrate in Fig 1A–1L wave propagation through an atrial sheet with varying ASD. Adrenergically stimulated sites enter the repolarization phase earlier than non-adrenergically stimulated sites, as indicated by scattered low-voltage elements amidst high-voltage elements. While there was no significant change in conduction speed across spatial densities, our observations are consistent with the AP signals shown in Fig 2A, where there is an overall decrease in APD, shortening the repolarization phase. We also observed a gradual development of an isopotential phase during repolarization of the atrial AP with increasing ASD.

Mean APD values decrease with increasing ASDs across all APD types as shown in Fig 4A, with mean *APD*_20_ values having the largest percentage change at 42% between 0% and 5%, while *APD*_50_ values changed only by 22%. Our analysis of GLMM results suggests that ASDs significantly affect all APD types. Furthermore, results from our pairwise comparison suggest that mean *APD*_20_ and *APD*_50_ values were significantly different across all spatial adrenergic densities (p < 0.005).

**Fig 3:**
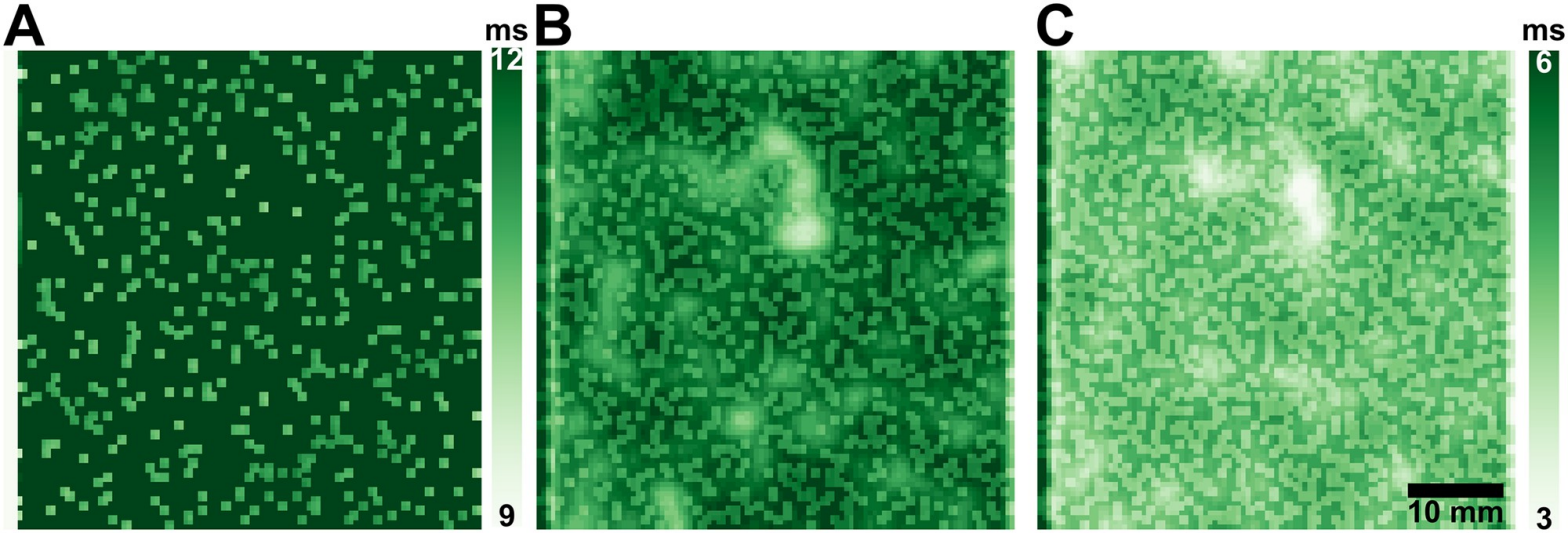
Spatial heterogeneity of *APD*_20_ due to increasing adrenergic spatial densities. Action potential duration (APD) maps of *APD*_20_ show that adrenergic spatial densities (ASD) at 5% (**A**), 10% (**B**), and 15% (**C**) increase overall spatial heterogeneity which could promote arrhythmogenicity in the atria. Note that only the APD maps of 10% and 15% are normalized between 3 ms and 6 ms while the APD map of 5% ASD is illustrated from 9 ms to 12 ms to demonstrate the shortening of *APD*_20_ due to increasing ASD.

**Fig 4:**
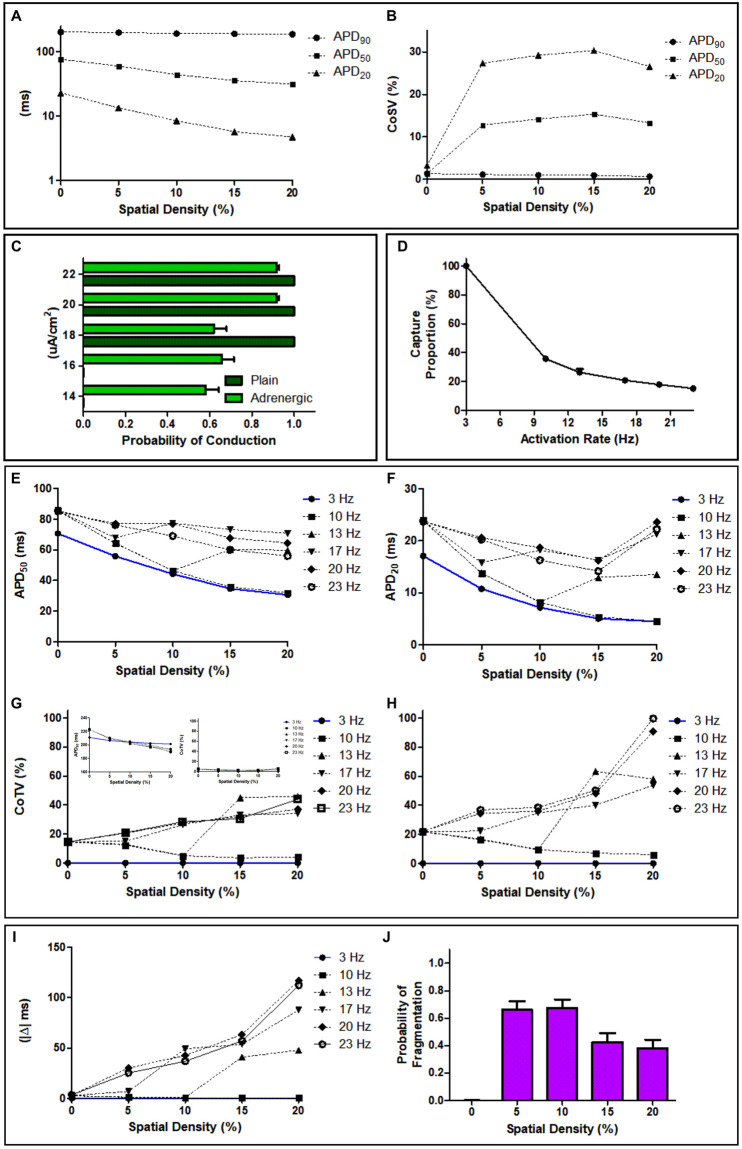
Measurement of the effects of varying adrenergic spatial densities, external transmembrane currents, and activation rates to Action Potential Durations (APD) and change to cycle lengths. (**A**)Sample log-scale distributions of *APD*_50_ and *APD*_20_ values across spatial densities of adrenergic stimulation of an atrial sheet. (**B**)Spatial heterogeneity of *APD*_50_ and *APD*_20_ values across spatial densities of adrenergic stimulation of an atrial sheet, measured as coefficient of spatial variation (CoSV). (**C**) Probability of conduction, or the likelihood of tissue excitation, due to changes in external transmembrane current with and without the presence of adrenergic elements across adrenergic spatial densities. The proportion of captured beats for increasing rapid activation rates in the presence of adrenergic elements (**D**). Effect of increasing activation rates to mean *APD*_50_ (**E**), and *APD*_20_ (**F**) values across spatial adrenergic densities. (**E inset**) Effect of activation rates to mean *APD*_90_ and its temporal heterogeneity. Our analysis of *APD*_90_ during increased activation is unremarkable nonetheless we included them for completion. Effect of increasing activation rates to temporal variation of *APD*_50_ (**G**), and *APD*_20_ (**H**) across spatial adrenergic densities. Effect of increasing adrenergic spatial densities to the absolute time difference (|Δ|) between the average cycle length of captured beats during rapid activation and pre-pacing cycle length of 300 ms(**I**). Probability of wave fragmentation, or the transformation of a uniform wavefront to single or multiple curvilinear ones, due to adrenergic spatial densities across activation rates (**J**).

Spatial heterogeneity measured with CoSV values reached their maxima at 15% for all APD types, as shown in Fig 4B. For the same spatial adrenergic density, *APD*_20_ has the largest CoSV value at 31% while CoSV value for *APD*_50_ is only 16%. We show *APD*_20_ maps of an atrial sheet with increasing ASD in Fig 3A–3C to visualize the spatial heterogeneity in APD due to ASD.

### Adrenergic spatial densities, excitation threshold, and activation rates

We showed in Fig 4C the probability of conduction for each external transmembrane current relative to increasing ASD. The excitation threshold for a plain 25 *cm*^2^ atrial sheet was 18 *μA*/*cm*^2^. However, adding a few adrenergic elements decreases our atrial sheets’ excitation threshold. We found that atrial sheets with at least 5% to 10% ASD could be excited with transmembrane currents below or equal 18 *μA*/*cm*^2^, with probabilities of conduction approximately 0.60 ± 0.07. However, atrial sheets with greater than 10% ASD are more likely to be activated at 20 *μA*/*cm*^2^ and above, with a probability of conduction greater than or equal to 0.92 ± 0.01. Table 1 visualizes the changes in excitation threshold with increasing ASD. Statistical analysis indicates significant conduction probability differences (p < 0.001) between external transmembrane currents.

**Table 1:** Effect of increasing adrenergic spatial density to the excitation threshold of an atrial sheet.

Adrenergic Spatial Densities and Excitation Threshold					
*μA*/*cm*^2^	0%	5%	10%	15%	20%
14		1			
16		1			
18	3	3	3		
20	3	3	3	3	3
22	3	3	3	3	3

Red-colored cells correspond to non-excitation, and green-colored cells correspond to excitation. The number of atrial sheets (out of 3) with observed conduction characteristics is shown within each cell.

The proportion of captured beats during rapid activation depends only on the activation rate, not ASDs. At 22 *μA*/*cm*^2^, we show in Fig 4D that increased activation rates decrease the proportion of captured beats for atrial sheets regardless of ASDs. While we report a one-to-one activation at 3 Hz, the proportion of beats captured during rapid activation for frequencies at 10 Hz and above range from 36% to 18%.

Similar to our analysis of *APD*_90_ in increasing ASD, *APD*_90_ results were unremarkable (Fig 4E inset). Therefore, we will focus our analysis on the *APD*_50_ and *APD*_20_. As we illustrated in Fig 2B, increasing ASD and activation rates above 10 Hz increases overall *APD*_50_ and *APD*_20_ values (Fig 4E and 4F). Mean *APD*_50_ and *APD*_20_ values of plain atrial sheets increase from 70 ms to 86 ms (21%) and from 17 ms to 24 ms (41%), respectively, when rapidly activated at 20 Hz. After adding 20% ASD, the mean *APD*_50_ increased from 30 ms baseline to 71 ms at 17Hz, and *APD*_20_ increased from 4 ms baseline to 24 ms at 20 Hz. Significant differences in mean *APD*_50_ (p < 0.001) and *APD*_20_ (p < 0.001) were observed across activation rates and ASDs.

Like their CoSV trend, *APD*_90_ values had the least CoTV, while *APD*_20_ values had the greatest among the APD types. We showed in Fig 4G and 4H CoTV of *APD*_50_, and *APD*_20_ across activation rates and ASDs. CoTV for *APD*_50_ values could reach up to 45% for 13 Hz and *APD*_20_ up to 100% for 23 Hz. Overall results from our statistical analysis showed a statistically significant difference in CoTV values across activation rates and ASDs for *APD*_50_ (p < 0.001) and *APD*_20_ (p < 0.001).

Activation times of captured beats vary with activation rate and ASDs. Fig 2C shows an example of activation time changes in captured beats during rapid activation. We illustrate in Fig 4I the absolute change (|Δ|) between the baseline activation cycle length and the cycle length of captured beats for increasing ASD as we increased activation rates. While increasing activation rates on a plain atrial sheet maintain similar baseline cycle lengths, increasing ASDs change the absolute cycle lengths of captured beats by 50 ms at 13 Hz and by as much as 120 ms at 23 Hz. Overall statistical analysis produced significant differences (p < 0.001) to the absolute activation time changes related to activation rates and ASDs.

**Fig 5:**
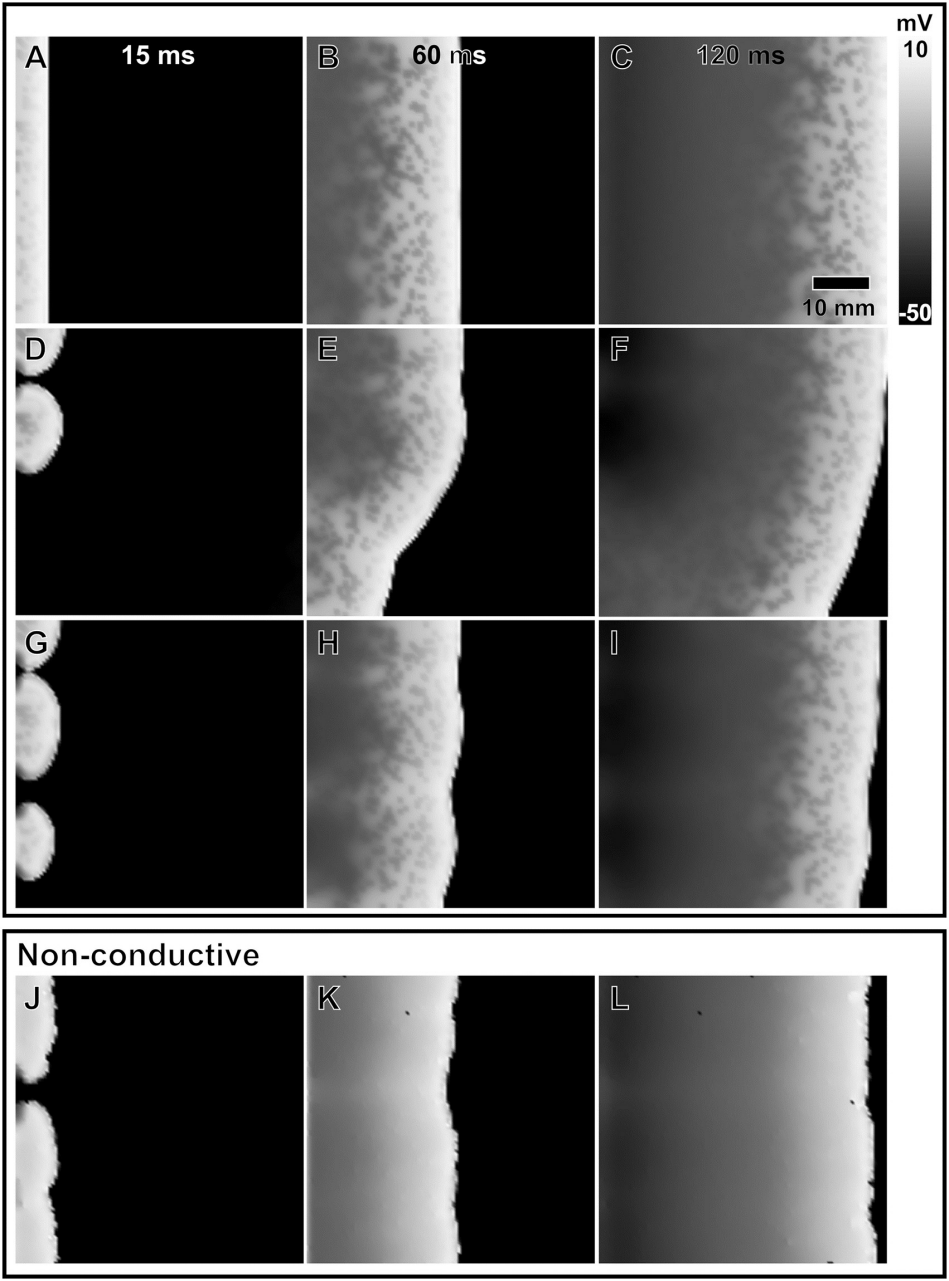
Wave fragmentation and propagation in a 10% adrenergically stimulated atrial sheets at three rates of rapid activation. Comparison of wave propagation in a 10% adrenergically stimulated atrial sheet at 10 Hz (**A**-**C**), 17 Hz (**D**-**F**), and 23 Hz (**G**-**I**). Comparison of a wave fragmentation and propagation in an atrial sheet with 10% non-conductive elements during rapid activation (**J**-**L**).

We illustrated in Fig 5 observed wave fragmentation events due to increased activation rate (i.e., 10 Hz at A-C, 17 Hz at D-F, and 23 Hz at G-I) for an atrial sheet with 10% ASD. We identified a fragmented wave as a transformed uniform wavefront to a single or multiple curvilinear one. While a captured beat at 10 Hz maintains a uniform wavefront profile across an adrenergically stimulated atrial sheet, captured beats at 17 Hz and 23 Hz cause the original uniform wavefront to be channeled at random entry points. Rapid activation in atrial sheets with adrenergic elements could fragment an otherwise uniform wavefront. We measured the probabilities of wave fragmentation due to ASD during rapid activation. We showed in Fig 4J that a uniform planar wave is most likely to be fragmented in rapidly activated atrial sheets with 10% ASD at 0.7 ± 0.06 likelihood. The probability of wave fragmentation decreases beyond 10%. The likelihood curve for wave fragmentation is highly correlated with the CoSV for *APD*_50_ (*ρ* = 0.87) and *APD*_20_ (*ρ* = 0.90) across ASDs. However only *APD*_20_ reached statistical significance (p = 0.04 vs. p = 0.06).

## Discussion

We presented the effects of varying ASDs on atrial sheets based on the Grandi atrial model. We simulated electrically remodeled human atrial sheets by gradually increasing the adrenergically stimulated elements. Furthermore, we simulated beta-adrenergic stimulation by changing ion channel conductances and affinities related to the beta-adrenergic response in atrial tissues, as prescribed in a previous work [23]. Based on our results, we could state the following:

- Increased ASDs could increase spatial heterogeneity of APDs in human atrial sheets;
- ASDs could change the excitation threshold of an atrial sheet;
- Rapidly activated atrial sheets with increased ASDs change different parts of the action potential, promote temporal variation, and influence the cycle length of captured beats;
- Increased ASDs could promote wave fragmentation, indicating a functional block.

We showed the shortening of atrial APD as ASD increases, consistent with findings from previous works [11, 32]. The shortening of atrial APD due to adrenergic stimulation has been attributed to excess intracellular *Ca*^2+^ due to the early release of *Ca*^2+^ from the sarcoplasmic reticulum, as shown in previous experimental [33–36] and simulation data [23, 32, 37, 38].

We showed that *APD*_50_ and *APD*_20_ values significantly decrease with increasing ASDs. While *APD*_90_, *APD*_50_, and *APD*_20_ are not typical indicators of arrhythmic tendencies, they have been used in previous animal and human experimental models to demonstrate electrophysiologic changes that may contribute to AF initiation [39–41]. Changes in *APD*_50_ and *APD*_20_ values could be partially explained by the incremental increase in AP amplitude and the appearance of a notch between an atrial AP’s depolarization and repolarization phases. The notch or isopotential phase halfway through repolarization results from a beta-adrenergic response to isoproterenol, as shown in a previous work [23].

Previous studies by Zhang et al. [9], Francoisi et al. [7], and Ripplinger et al. [6] suggested that differences in spatial densities of adrenergic stimulation could increase the heterogeneity of conduction and repolarization to promote arrhythmogenic substrates. Since we kept longitudinal and transverse conductivity values uniform, we kept conduction velocities consistent for all atrial sheet preparations. Therefore, we demonstrated that changes in ASDs resulted in varying degrees of atrial repolarization heterogeneity in isolation. Our simulation results suggest that changes in the spatial distribution of sympathetic neuroeffector junctions on the atria could be pro-arrhythmic. Moreover, spatial heterogeneity of repolarizations could be maximized even in only partially innervated, adrenergically stimulated atrial tissues.

We also examined the role of adrenergic elements in the likelihood of conduction in atrial sheets. An early work by Vicenzi and West in various animals showed that direct sub-threshold stimulation of left atria is sufficient to release autonomic mediators (i.e., norepinephrine and acetylcholine) from the sinoatrial node but not enough to excite the myocardium [42]. Vicenzi added that their observations could be related to the heterogeneous distribution of neural elements (i.e., neuroeffector junctions, tracts, and ganglia) within the myocardium. Vicenzi and West supplemented their findings in a subsequent paper, promoting the release of autonomic mediators from the sinoatrial node by modifying intracellular calcium [43]. Our work supports their early observations by demonstrating that the heterogeneous distribution of neural elements may contribute to the release of autonomic mediators and lower the excitation threshold of atrial tissues. Furthermore, our study extends Vicenzi and West’s work by showing that autonomic mediators that promote adrenergic effects potentially play a role in changing excitation thresholds.

Fast activation rates in densely innervated, adrenergically stimulated atrial substrates may be pro-arrhythmic. *APD*_20_ values have the greatest temporal variation, followed by *APD*_50_ values, for rapidly activated atria with spatially dense adrenergic elements. Moreover, as we showed in Fig 2C, mean activation times drastically change with increasing activation rate. While desensitization of adrenergic receptors due to prolonged hyperactivity is more common in heart failure [44, 45] and aging patients [46], our current work aims to replicate atrial tissues from young patients who are less likely to have remodeled substrate. Our simulations show that despite the potential desensitization of adrenergic receptors during sympathetic hyperactivity, wave fragmentation could still occur with only 5% ASD within two seconds of rapid activation.

Our study shows the potential electrophysiological contribution of adrenergic spatial densities to atrial arrhythmias caused by sympathetic hyperactivity. The increased spatial and temporal heterogeneity due to increased ASDs could mimic a functional block, which results in wave fragmentation, as demonstrated in Fig 5. Sharifov et al. [47] showed that wave fragmentation due to autonomic mediation of the atria could result in AF in canine models. Sharifov’s team posited that wave fragmentation due to autonomic mediation could be explained by transient inexcitability near the pacemaker region. As illustrated in Fig 5D and 5G, wave fragments initiate near the stimulation source where transiently inexcitable elements could be located. Due to the changes in APD characteristics of adrenergically stimulated atrial locations, some elements of our atrial sheet in the immediate area of stimulation may have different refractory periods compared to non-adrenergic elements. To support our observations, we show that the probabilities of wave fragmentation are highly correlated with the spatial variation of *APD*_20_ due to heterogeneous adrenergic stimulation (shown in Fig 3). While the spatial variation of *APD*_50_ is also highly correlated to the likelihood of wave fragmentation, it did not reach statistical significance. We could attribute their correlations to ASDs since we also showed that wave fragmentation due to non-conductive elements occurs at higher activation rates and has different fragmentation patterns than adrenergically stimulated sheets. However, *APD*_20_ has not been concretely associated with the atrial refractory period and would require further investigation. While Sharifov et al. [47] examined acetylcholine-mediated AF, spatial adrenergic densities share similarities in promoting arrhythmogenic substrates. Our current study could support the idea of the autonomically-mediated functional block in the human atria, as found in previous animal models.

Our work is similar to Celotto et al.’s study, which demonstrated the effects of spatially distributed parasympathetic activation on a two-dimensional fibrotic atrial sheet [48]. Celotto et al. showed an overall shortening of action potential repolarization at locations stimulated by acetylcholine, which affected the overall morphology of electrograms from virtual electrodes. Moreover, a simulation study by Muñoz et al. showed the role of heterogeneous cholinergic activation on three-dimensional rabbit atrial models [49]. Muñoz et al. demonstrated that a re-entry triggered by the sinoatrial node could result from a heterogeneous cholinergic response, large myocardial load, and extensive innervation of the sinoatrial node. We extend the works of Celotto et al. and Muñoz et al. by isolating the role of ASD in creating arrhythmogenic atrial substrates during sympathetic hyperactivity. In addition, our work provides a possible electrophysiologic link for human atrial arrhythmia due to hyperactivity of sprouted sympathetic nervous tissues as previously observed by Chang et al. [15] and Akira et al. [16] in animal models.

### Limitations

While more comprehensive and intricate models for adrenergic stimulation of the human myocardium have been introduced [37, 38], the Grandi atrial model is sufficient to demonstrate the effects of ASDs without the additional variability from isoproterenol dosage and increased computational loads from more detailed ionic models. However, we recognize that adrenergic stimulation is not a binary event, and different levels may influence arrhythmogenicity in the atria.

We recognize that alpha-adrenergic receptors are also present in the human myocardium; however, the Grandi atrial model assumes that adrenergic effects could be modeled with only beta-adrenergic activity. Electrophysiologic changes due to sympathetic hyperactivity could be logically attributed primarily to beta-adrenergic activity since alpha-adrenergic effects do not affect conduction and refractoriness [50]. Moreover, alpha-adrenergic effects are better associated with blood pressure regulation due to increased vagal tone [29, 51, 52]. Finally, alpha-adrenergic receptors are scarce in the human myocardium as shown in previous studies [27, 28] and play a protective role only during heart failure.

We acknowledge that we modeled only atrial sheets, so our simulations do not account for the three-dimensional properties of tissues. However, similar to the work of Celotto et al. [48], we showed that an atrial sheet is also sufficient to study autonomic stimulation of the myocardium. We also acknowledge that we did not model different patterns of adrenergic densities, similar to the work by Celotto et al. [48]. However, sympathetic nerve sprouting in animal models simulating hyperactivity does not appear to have specific organization or patterns [14–16]. Zhu et al. also showed that sympathetic neuroeffector junctions over myocardial tissues are spatially heterogeneous [5]. Therefore, we simulated atrial sheets to be adrenergically stimulated at random locations to systematically isolate the role of adrenergic spatial densities in initiating atrial arrhythmias.

Finally, we recognize that we focused on adrenergic stimulation of atrial substrates to simulate the effects of sympathetic hyperactivity. While previous works have established that sympathetic overdrive and parasympathetic withdrawal are indicators of autonomic dysfunction due to chronic stress [6, 53], wave fragmentation as a result of sympathetic hyperactivity has not been characterized through increased and dispersed ASD in human atrial substrates in silico.

## Conclusion

Sympathetic hyperactivity due to autonomic dysfunction could promote diffuse sympathetic nerve sprouting and create arrhythmogenic atrial substrates. We demonstrated, via simulations, that human atrial sheets with varying adrenergic spatial densities increase the spatial and temporal heterogeneities of APDs, characteristic of arrhythmic vulnerability. Low adrenergic spatial densities decrease atrial sheets’ activation threshold; however, the activation threshold increases in atrial sheets with high adrenergic spatial densities. With rapid activation, high adrenergic spatial densities result in prolonged durations and increased temporal variations of *APD*_50_ and *APD*_20_. Lastly, our simulations suggest that the presence of adrenergic elements during rapid activation could cause wave fragmentation due to transient inexcitability and is highly correlated with the spatial heterogeneity of atrial APDs. Our work may aid in elucidating the contribution of hyperactive and diffuse sprouted sympathetic nerves and human atrial arrhythmias. Our study may provide a possible electrophysiological link between sympathetic hyperactivity and the initiation of paroxysmal or lone AF among chronically stressed individuals without substrate remodeling.

## Supporting information

S1 Data (XLSX)
